# The Macrophage Inhibitor CNI-1493 Blocks Metastasis in a Mouse Model of Ewing Sarcoma through Inhibition of Extravasation

**DOI:** 10.1371/journal.pone.0145197

**Published:** 2015-12-28

**Authors:** Anthony J. Hesketh, Caroline Maloney, Christopher A. Behr, Morris C. Edelman, Richard D. Glick, Yousef Al-Abed, Marc Symons, Samuel Z. Soffer, Bettie M. Steinberg

**Affiliations:** 1 The Elmezzi Graduate School of Molecular Medicine, North Shore-LIJ Health System, Manhasset, New York, United States of America; 2 Center for Oncology and Cell Biology, The Feinstein Institute for Medical Research, North Shore-LIJ Health System, Manhasset, New York, United States of America; 3 Department of Surgery, Hofstra North Shore-LIJ School of Medicine, New Hyde Park, New York, United States of America; 4 Department of Pathology and Laboratory Medicine, Hofstra North Shore-LIJ School of Medicine, New Hyde Park, New York, United States of America; 5 Center for Molecular Innovation, The Feinstein Institute for Medical Research, North Shore-LIJ Health System, Manhasset, New York, United States of America; 6 Department of Molecular Medicine, Hofstra North Shore-LIJ School of Medicine, Manhasset, New York, United States of America; University of Alabama at Birmingham, UNITED STATES

## Abstract

Metastatic Ewing Sarcoma carries a poor prognosis, and novel therapeutics to prevent and treat metastatic disease are greatly needed. Recent evidence demonstrates that tumor-associated macrophages in Ewing Sarcoma are associated with more advanced disease. While some macrophage phenotypes (M1) exhibit anti-tumor activity, distinct phenotypes (M2) may contribute to malignant progression and metastasis. In this study, we show that M2 macrophages promote Ewing Sarcoma invasion and extravasation, pointing to a potential target of anti-metastatic therapy. CNI-1493 is a selective inhibitor of macrophage function and has shown to be safe in clinical trials as an anti-inflammatory agent. In a xenograft mouse model of metastatic Ewing Sarcoma, CNI-1493 treatment dramatically reduces metastatic tumor burden. Furthermore, metastases in treated animals have a less invasive morphology. We show *in vitro* that CNI-1493 decreases M2-stimulated Ewing Sarcoma tumor cell invasion and extravasation, offering a functional mechanism through which CNI-1493 attenuates metastasis. These data indicate that CNI-1493 may be a safe and effective adjuvant agent for the prevention and treatment of metastatic Ewing Sarcoma.

## Introduction

Ewing Sarcoma is the second-most common bone and soft tissue malignancy of childhood. One in four patients have detectable metastases at the time of diagnosis. The long-term prognosis for patients with metastatic Ewing Sarcoma is poor, as more than two-thirds of these children will succumb to their disease within five years [1]. Multi-agent chemotherapy is the mainstay of treatment, complemented by surgery and radiation for local control of the primary tumor site or isolated metastatic disease. Although these modalities may be successful for limited, local disease, metastatic Ewing Sarcoma continues to be a great challenge with few effective treatments. Various strategies, ranging from antiangiogenic therapy to myeloablative “megatherapy” with autologous stem cell rescue, have not resulted in an effective, reproducible and standardized approach to controlling advanced disease [2]. New therapeutic options are urgently needed to address these high-risk patients.

Macrophages are phagocytic derivatives of circulating bone marrow-derived monocytes. Upon infiltration into tissues, macrophages serve a variety of homeostatic and immunoregulatory roles important to the adaptive and innate inflammatory responses. Similar to other immunoregulatory cells, macrophages exist in a spectrum of functional states, the extremes of which can be generally characterized by the pro-inflammatory (M1) and anti-inflammatory (M2) phenotypes [3]. Macrophages that infiltrate and subsequently comprise a substantial portion of the tumor microenvironment are termed tumor-associated macrophages (TAMs). Macrophages are recruited to the tumor microenvironment where they consequently adapt an M2-like phenotype supportive of tumor growth [4–7], although details of the origin and features of this phenotype have recently been debated [8]. In this state, macrophages promote angiogenesis, cellular proliferation, viability, motility and invasion, tissue remodeling, and immune suppression [4, 6, 9–18]. This TAM-induced malignant progression is reflected by a number of studies on various human tumor specimens that correlate overexpression of macrophage chemoattractants, as well as increased numbers of infiltrating macrophages, with worse prognoses [19, 20]. There is also increasing evidence that macrophages play an important role in establishing the “premetastatic niche,” altering the stromal environment at sites far from the primary tumor and enhancing extravasation and growth of metastatic cells [21–25]. Few studies have examined the role of macrophages in Ewing Sarcoma. One study of 76 primary tumor specimens demonstrated an inverse relationship between TAM content and patient survival [26]. Although the evidence is limited, this relationship suggests that TAMs may promote Ewing Sarcoma disease progression.

Presently, several TAM-directed cancer immunomodulatory agents are being developed, with some reaching clinical trials [10, 27–34]. CNI-1493, also known as semapimod, is a small molecule anti-inflammatory agent that has been shown to inhibit production of macrophage-derived inflammatory mediators without significantly affecting other cell lineages [35–37]. Initially conceptualized as an inhibitor of arginine transport, its mechanism remains unclear: cytokine suppression is seen at concentrations far below those required for inhibition of arginine transport [38–40]. Although the mechanism of action remains to be delineated, CNI-1493 has been deemed safe and well tolerated in humans, having completed a phase II clinical trial for Crohn’s disease without significant adverse side effects [41–44]. Given its safety profile, CNI-1493 has recently been identified as a possible TAM-targeting antitumor agent. CNI-1493 treatment in mouse models of breast cancer and glioblastoma has demonstrated an ability to decrease tumor invasion and rate of metastasis [45, 46].

In this study, we find that CNI-1493 markedly decreases the incidence of invasive metastasis and tumor burden in a mouse model of Ewing Sarcoma, and that it suppresses M2 macrophage-stimulated tumor cell invasion and extravasation *in vitro*.

## Materials and Methods

### Ethics Statement

Studies involving the use of human primary cells were approved by the Institutional Review Board at the North Shore-LIJ Health System. Written informed consent was given by blood donors.

This study was carried out in strict accordance with the recommendations in the Guide for the Care and Use of Laboratory Animals of the National Institutes of Health. The protocol was approved by the Institutional Animal Care and Use Committee of the Feinstein Institute (Permit Number: 2005–060). All surgery was performed under anesthesia, and all efforts were made to minimize suffering.

### Reagents

CNI-1493 was produced in-house by Dr. Yousef Al-Abed and prepared as a stock solution of 20 mM in 7% DMSO. It was diluted for animal and *in vivo* experiments to working concentrations with PBS, with final DMSO concentrations of 0.4 and 7x10^-5^ percent, respectively.

Antibodies with the following specificities and concentrations were used in this study: mouse monoclonal CD3-Pacific Blue (5 μl per 10^6^ cells, RRID:AB_493095, BioLegend Cat# 300418, San Diego, CA, USA), mouse monoclonal CD14-PE (10 μl per 10^6^ cells, RRID:AB_357169, R&D Systems Cat# FAB3832P, Minneapolis, MN, USA) and mouse monoclonal unconjugated CD99 (1:50, RRID:AB_2076419, Dako Cat# M3601, Carpinteria, CA, USA).

### Cell Culture

The human Ewing Sarcoma cell line SK-NEP-1 [47, 48] was purchased from ATCC (HTB-48, Manassas, VA, USA). The established human Ewing Sarcoma cell lines CHLA-10 [49–51], CHLA-32 [50] and TC-71 [50–53] were generously provided by the Children’s Oncology Group Cell Culture and Xenograft Repository (COGcell.org) which is supported by the Alex’s Lemonade Stand Foundation. Ewing Sarcoma cell lines were obtained directly from specified sources, maintained in a low passage and are not listed in the ICLAC Database of Cross-contaminated or Misidentified Cell Lines. Expression of the EWS/FLI1 fusion gene characteristic of most Ewing Sarcomas has been previously confirmed in each cell line used [47, 54]. All Ewing Sarcoma cell lines were cultured in complete RPMI consisting of RPMI-1640 (HyClone Laboratories, Logan, UT) with 10% heat inactivated fetal bovine serum (FetalClone II, Hyclone Laboratories) and 1% penicillin/streptomycin (HyClone Laboratories). Human peripheral blood mononuclear cells were isolated from the blood of healthy donors by 1:1 dilution of whole blood in Dulbecco’s phosphate buffered saline (PBS), centrifugation on a Ficoll-Paque PLUS (GE Healthcare Bio-Sciences, Pittsburgh, PA, USA) gradient and extraction of the mononuclear cell layer. Cells were washed and centrifuged to remove platelets and magnetic-activated cell sorting enriched for CD14^+^ monocytes using the Monocyte Isolation Kit II (Miltenyi Biotec Inc., San Diego, CA, USA) according to the manufacturer’s instructions. In some cases, pre and post-sorting aliquots of 1x10^5^ cells were used for flow cytometry analysis to evaluate monocyte purity. Cells were stained with CD3-Pacific Blue and CD14-PE at 37°C for 30 minutes. Flow cytometry was performed with a FACSVerse flow cytometer (BD Biosciences, San Jose, CA, USA) and the data analyzed with FlowJo software (V. 10.0.7r2, FlowJo LLC, Ashland, OR, USA). CD14^+^ monocytes represented greater than 90% of the cell population used for further macrophage derivation (S1 Fig).

Sorted monocytes were grown in complete RPMI supplemented with 25 ng/ml human GM-CSF or M-CSF (BioLegend) for seven days to polarize to M1 or M2 macrophages, respectively. Supplemented complete media was changed on the third and sixth days. After seven days, polarized macrophages were activated to M1s by supplementation with 20 ng/ml human recombinant interferon-γ (PeproTech, Rocky Hill, NJ, USA) and 100 ng/ml lipopolysaccharide from *Escherichia coli O111*:*B4* (InvivoGen, San Diego, CA, USA) for 18 hours. Alternatively, macrophages were activated to M2s by supplementation with human recombinant interleukin-4 (PeproTech) [55–60]. Macrophages were treated with 200 nM CNI-1493 or vehicle during activation steps and subsequent experiments.

Human Pulmonary Microvascular Endothelial Cells (HPMECs) were kindly provided by Dr. Ronald Unger from the Institute of Pathology, Johannes Gutenberg University as HPMEC-ST1.6R and used under the supervision of Dr. Edmund Miller [61]. This cell line is not listed in the ICLAC Database of Cross-contaminated or Misidentified Cell Lines. HPMECs were cultured in Endothelial Cell Media (containing 5% fetal bovine serum, 1% endothelial cell growth supplement and 1% penicillin/streptomycin)(ScienCell Research Laboratories, Carlsbad, CA, USA). At least 48 hours before experimentation with HPMECs, media was changed to RPMI with 10% fetal bovine serum and 1% penicillin/streptomycin. All cell cultures were grown at 37°C in a humidified atmosphere of 5% CO_2_, 95% air.

### Microarray Analysis

Immediately following activation and treatment with CNI-1493 or vehicle, cells from three separate anonymous donors were lysed and their RNA extracted with the High Pure RNA Isolation Kit (Roche Diagnostics, Indianapolis, IN, USA). RNA amplification and cRNA synthesis was completed using the Ambion Illumina TotalPrep RNA Amplification Kit (Life Technologies, Grand Island, NY, USA). Labeled cRNA was hybridized to HumanHT-12v4 Expression BeadChips (Illumina Inc., San Diego, CA, USA) and washed and scanned according to the IntelliHyb Seal protocol (Illumina). The chip was scanned on a HiScan Array Scanner (Illumina) and images processed with BeadStudio (Illumina). Signal intensities were cubic spline normalized with background subtraction in GenomeStudio Gene Expression Analysis Module (V. 1.9.0) within the GenomeStudio environment (V. 2011.1, Illumina). Data were also analyzed using the Genesifter environment (VizX Labs, Seattle, WA, USA). Raw and cubic spline-normalized data from the arrays are available from the Gene Expression Omnibus (GEO) database [62] and are accessible through GEO Series accession number GSE66805 (http://www.ncbi.nlm.nih.gov/geo/query/acc.cgi?acc=GSE66805). Differentially expressed genes were cross-referenced with 97 previously published genes that reflect M1/M2 polarization [63]. Sixty-one differentially expressed genes were row-normalized and heatmaps were generated using the PreprocessDataset (V. 5) and HeatMapViewer (V. 13) modules within the GenePattern analysis environment (V. 2.0, Broad Institute, Cambridge, MA, USA) [64].

### Invasion Assays

Invasion assays were modified from previously published protocols [45, 65, 66]. Briefly, SK-NEP-1 cells labeled with CellTracker Green CMFDA dye (Life Technologies) and M1- or M2-polarized macrophages were embedded in 50 μl of 10 mg/ml Cultrex basement membrane extract (BME) (Trevigen, Gaithersburg, MD, USA) on ice. CHLA-10, CHLA-32 and TC-71 cells were similarly embedded in BME with M1- or M2-polarized macrophages.BME mixtures containing tumor cell/macrophage co-cultures were then placed into the bottom of 24-well fluorescent-opaque transwell inserts (Corning Incorporated Life Sciences, Tewksbury, MA, USA). Transwell inserts were previously coated with 1 μg/ml fibronectin on the bottom side of the 8 μm filter and allowed to polymerize at 30 minutes at 37°C. Cells were plated at a density of 5x10^4^ polarized macrophages and 1x10^5^ Ewing Sarcoma cells per invasion chamber. Serum-free RPMI was added to both wells, and 200 nM CNI-1493 or its diluent was added into the BME and in the media above and below the transwell. Invasion chambers were incubated for 72 hours. Fluorescent tumor cells adherent to the bottom of the transwell filter were viewed with an Olympus IX70 inverted fluorescence microscope (Olympus America, Melville, NY, USA) and manually counted.

### Proliferation Assays

SK-NEP-1 cells were transduced with a Red-FLuc-GFP lentiviral vector under control of the UbC promoter (Targeting Systems, El Cajon, CA, USA). GFP^+^ SK-NEP-1 cells starved of serum for 48 hours were stained with CellTrace Violet (Life Technologies) according to the manufacturer’s instructions and then cultured alone or with freshly polarized M1 or M2 macrophages in a ratio of 2:1. Cultures were treated with CNI-1493 or vehicle both in the presence of 10% fetal bovine serum and in serum-free media. After 96 hours, cells were washed, gently scraped and resuspended in a single cell suspension. Cells were analyzed by flow cytometry on an LSRFortessa (BD Biosciences) and violet fluorescence intensity measured for GFP^+^-gated cells. Post-acquisition analysis was performed with FlowJo software (V. 10.0.7r2, FlowJo LLC).

### Animal Studies

Six week-old female nude NCr mice (Taconic Biosciences, Germantown, NY, USA) were inoculated in the left kidney with SK-NEP-1 cells. Animals were anesthetized with ketamine (50 mg/kg) and xylazine (5 mg/kg) and the left kidney was exposed through a left flank incision. An inoculum of 10^6^ SK-NEP-1 tumor cells in 0.1 ml of PBS was injected into the renal capsule using a 25-gauge needle. Musculofasciae were closed with 5–0 Vicryl sutures and skin incisions closed with staples. In the first replicate experiment, mice (n = 18) were implanted with a 3 cm osmotic pump (ALZET Osmotic Pumps, Cupertino, CA, USA) placed within a subcutaneous pocket at the site of the skin incision, with a catheter (ALZET Osmotic Pumps) placed through the musculofascial incision into the peritoneal cavity for intraperitoneal drug delivery. Osmotic pumps were previously filled and primed with CNI-1493 or vehicle to deliver 5 mg/kg/day of drug or equal volume diluent according to the manufacturer’s instructions. Mice were randomized to receive drug or vehicle. In the second replicate, mice (n = 28) were randomized to receive daily intraperitoneal injections of 5 mg/kg CNI-1493 or equal volume diluent beginning seven days after tumor cell inoculation.

Six weeks after beginning treatment, mice were sacrificed and primary tumors and lungs were harvested, weighed and preserved in 4% paraformaldehyde. Paraffin-embedded tumors and lungs were cut into 4 μm sections using four levels from each lung, then stained by routine hematoxylin and eosin methods. Slides were reviewed with a pediatric pathologist in a blinded fashion and morphologies of the metastases were categorized based upon their presence of invasion into lung parenchyma. Immunohistochemistry for CD99 was performed on adjacent sections to confirm metastatic foci of Ewing Sarcoma. Negative controls for CD99 immunohistochemistry were established on adjacent lung sections by omitting incubation with CD99 primary antibody and did not demonstrate nonspecific binding. Micrographs were acquired on a Zeiss Axiovert 200M inverted microscope (Carl Zeiss AG, Oberkochen, Germany) with AxioVision software (V. 4.8.2.0, Carl Zeiss AG). Total surface area of metastatic foci per lung section from resulting images was measured using ImageJ (V. 1.47, National Institutes of Health, Bethesda, MD, USA).

### Gelatinase Assay

SK-NEP-1 cells and M1 or M2 macrophages were cultured alone or in tumor cell-macrophage co-culture in a ratio of 2:1 and treated with 200 nM CNI-1493 or vehicle for 48 hours. Supernatants were collected and concentrated using 156 kDa centrifugal filters (Amicon Ultra-15 Centrifugal Filter Units, EMD Millipore, Darmstadt, Germany). Cells were collected and lysed with TNE buffer without protease inhibitor. Concentrated supernatant and cell lysates were assayed for gelatinase activity using the EnzChek Gelatinase Assay Kit (Life Technologies). Briefly, protein concentrations of samples were determined using a BCA assay (Thermo Fisher Scientific Inc., Rockford, IL, USA) and volumes equaling 30 μg were incubated with fluorescein-conjugated DQ-gelatin in a 96-well plate for one, two and 24 hours at 20° protected from light. Serial dilutions of collagenase from *Clostridium histolyticum* and reaction buffer were also incubated with DQ-gelatin as standards and negative control. Fluorescence intensity at each time point was measured with a GENios Pro fluorescent spectrophotometer (Tecan Group Ltd., Männedorf, Switzerland) with a standard fluorescein filter. Resulting data were calculated in equivalents of *C*. *histolyticum* units after subtraction of background fluorescence determined from the no-enzyme control.

### Extravasation Assays

Extravasation assays were performed as a modification of the previously specified invasion assays and as described in the literature [67]. Briefly, HPMECs were cultured on a layer of BME coating the surface of fluorescence-opaque transwell culture inserts (BD BioCoat Tumor Invasion System)(BD Biosciences) that had previously been coated with 1 μg/ml fibronectin on the undersurface of the 8 μm filter. RPMI supplemented with 200 nM CNI-1493 or vehicle was added to the upper and lower chambers. As controls, HPMECs were not present in a subset of wells to determine invasion in the absence of an endothelial monolayer. After 24 hours, an HPMEC monolayer was confirmed with light microscopy. Permeability of the monolayer was verified by adding 1% Evans Blue dye (Thermo Fisher Scientific, Inc.) to the upper chamber, followed by measurement with a spectrophotometer (SpectraMax 340, Molecular Devices LLC, Sunnyvale, CA, USA) at 610 nm of media in the lower chamber after one hour (S2 Fig). 1x10^5^ transduced, GFP^+^ SK-NEP-1 cells alone or in co-culture with 5x10^4^ M2-polarized macrophages were placed in the upper chamber. Invasion chambers were incubated for 72 hours and fluorescent cells adherent to the undersurface of the transwell filter were viewed with an Olympus IX70 inverted fluorescence microscope (Olympus America) and counted.

### Statistics

For animal studies, tumor weights were reported as mean ± standard deviation. Pulmonary metastatic tumor burden as determined by surface area of metastatic foci or invasive metastatic foci per lung section per mouse were reported as mean ± standard deviation. Unpaired, two-tailed *t* tests with Welch’s correction were used to evaluate the difference in primary tumor size and metastatic tumor burden. Two-tailed Fisher’s exact tests were used to evaluate incidence of metastasis or invasive metastasis. For *in vitro* studies, two-way analyses of variance (ANOVA) with post hoc Tukey’s multiple comparisons tests were utilized to determine differences in invasion and extravasation assays. Statistical analyses were performed using Prism (V. 6.0c, GraphPad Software Inc., La Jolla, CA, USA). A p-value <0.05 was considered statistically significant.

## Results

### CNI-1493 decreases human M2-induced Ewing Sarcoma tumor cell invasion in vitro

**Fig 1:**
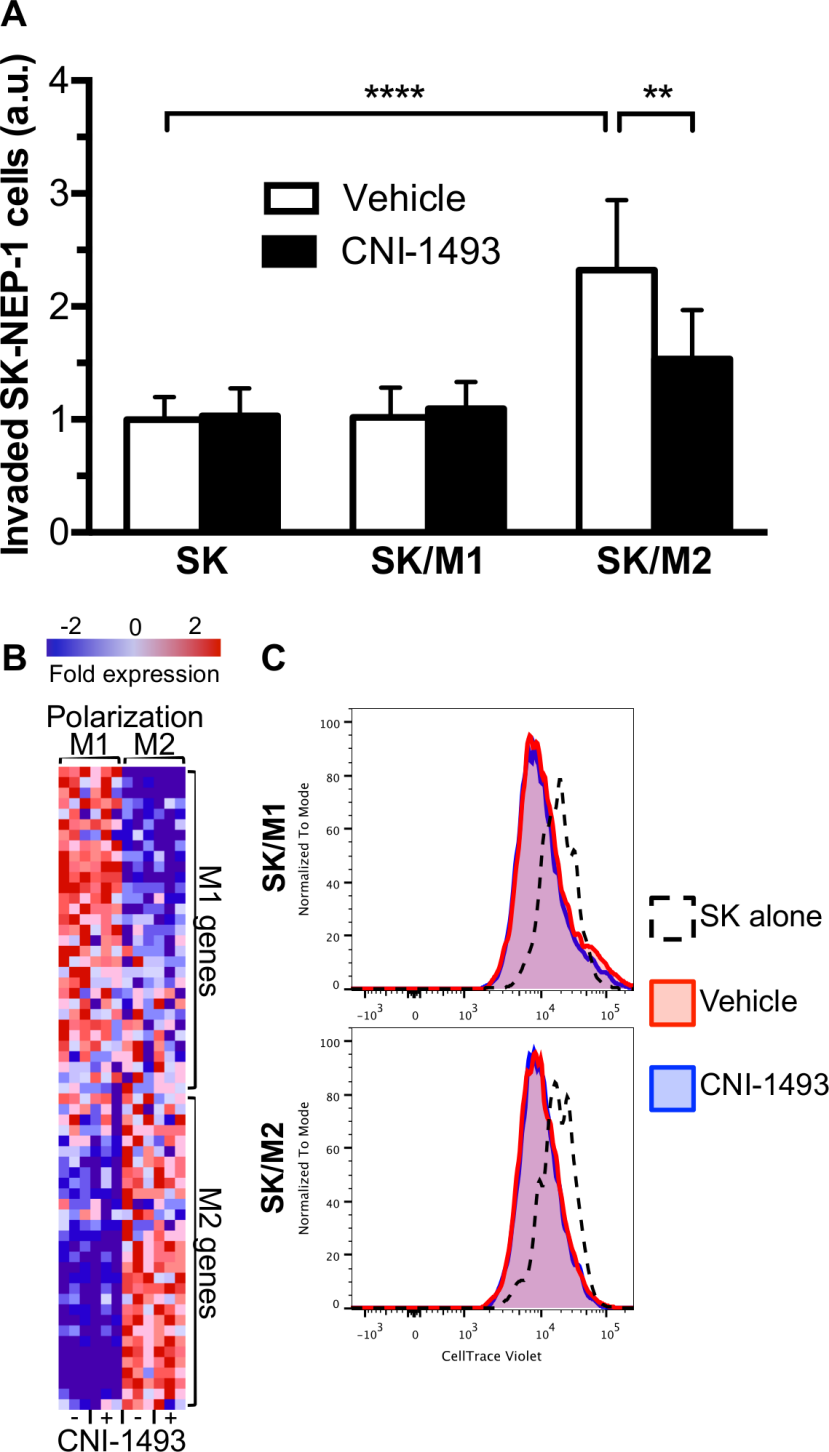
M2-induced invasion of Ewing Sarcoma tumor cells is inhibited by CNI-1493. (A) Fluorescently labeled SK-NEP-1 cells were cultured alone (SK) or in co-culture with M1- or M2-polarized macrophages (SK/M1 or SK/M2, respectively) in basement membrane extract within a transwell invasion culture system. Transwell systems were treated with 200 nM CNI-1493 or vehicle and the number of invading cells were counted after 72 hours. Arbitrary units represent fold change of invaded cells in vehicle-treated SK-NEP-1-only wells. **p<0.01, ****p<0.0001. Bars represent mean ± standard deviation of three experiments. (B) Gene microarray was performed on RNA extracted from M1 and M2 macrophages treated with 200 nM CNI-1493 or vehicle. Differentially expressed genes were cross-referenced with 97 previously published M1 and M2 genes as described by Martinez et al [63]. Sixty-one genes were identified and represented as a heatmap with gene expression data normalized to row. Gene names are listed in S1 Table. (C) Proliferation assay of SK-NEP-1 cells cultured alone or in co-culture with M1 or M2 macrophages (SK/M1 or SK/M2, respectively). SK-NEP-1 cells were transduced with a GFP expression vector and stained with CellTrace Violet. Cultures were treated with 200 nM CNI-1493 or vehicle and maintained in the absence of serum for 96 hours. GFP^+^ cells were then FACS analyzed for intensity of CellTrace Violet staining.

**Fig 2:**
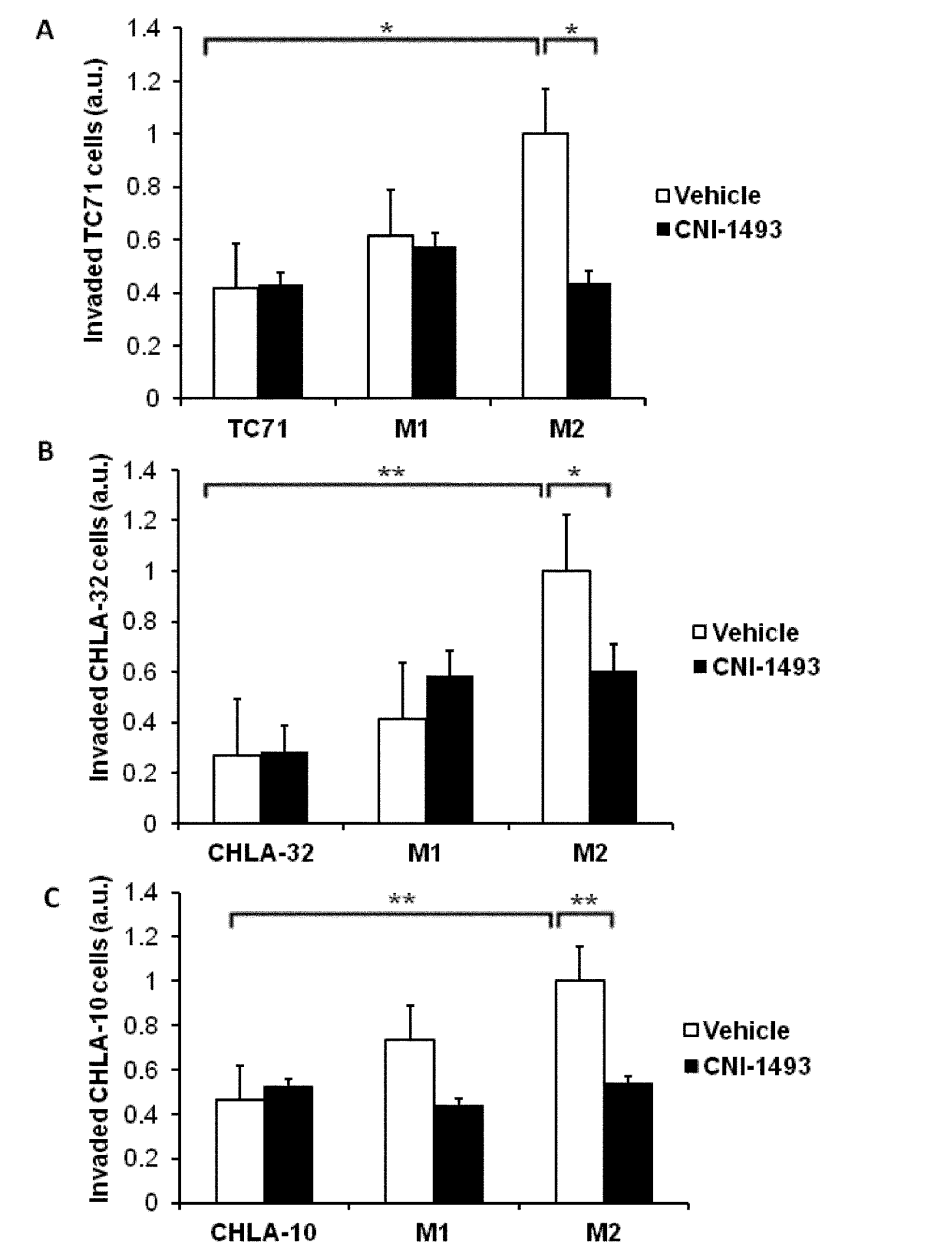
M2-induced invasion of Ewing Sarcoma tumor cells is inhibited by CNI-1493 in multiple cell lines. Fluorescently labeled TC71, CHLA-32, or CHLA-10 cells were cultured alone or in co-culture with M1- or M2-polarized macrophages (labeled M1 or M2) in basement membrane extract within a transwell invasion culture system. Transwell systems were treated with 200 nM CNI-1493 or vehicle and the number of invading cells were counted after 72 hours. Arbitrary units represent fold change of invaded cells in vehicle-treated SK-NEP-1-only wells. *p<0.05, **p<0.001. Bars represent mean ± standard deviation of three experiments.

We first asked whether human primary M1 and M2 macrophages derived from blood monocytes altered invasion of Ewing Sarcoma cells through a basement membrane extract using three-dimensional invasion assays, and whether CNI-1493 altered that process (Fig 1A). SK-NEP-1 cells alone had little capacity to invade, which was unchanged when co-cultured with M1 macrophages. In contrast, M2 macrophages significantly increased tumor cell invasion (p<0.0001), and this stimulation was suppressed by CNI-1493 (p<0.01). CNI-1493 had no effect on invasion by SK-NEP-1 cells alone or when co-cultured with M1 macrophages, indicating that CNI-1493 was not having a direct effect on tumor cells or a generalized effect on all macrophages. To assure that these results were not unique to SK-NEP-1 cells, stimulation of Ewing Sarcoma tumor cell invasion by M2 macrophages and decrease in M2-induced tumor cell invasion by CNI-1493 treatment was confirmed in three additional cell lines, CHLA-10, CHLA-32 and TC-71 (Fig 2)

Gene microarray analysis of macrophages treated with CNI-1493 demonstrates that the gene expression pattern of M1 and M2 cells does not change (Fig 1B), indicating that CNI-1493 is not simply inducing a switch in macrophage phenotypes, conferring the properties of M1 macrophages on previously polarized M2 macrophages. Tumor-associated macrophages have been demonstrated to increase tumor cell proliferation, serving as a source of growth factors and promoting tumor cell survival. As seen in Fig 1C, both M1 and M2 macrophages stimulate proliferation of SK-NEP cells in serum-free medium. A subpopulation of tumor cells did not proliferate in the presence of M1 macrophages, as demonstrated by a positive skew in the fluorescence intensity histogram. CNI-1493 had no effect on M1- or M2-induced tumor cell proliferation. Therefore, the M2-induced increase in tumor cell invasion shown in Fig 1A was not simply due to increased number of tumor cells, and CNI-1493’s suppression was not a result of cell loss. Finally, tumor-associated macrophages can support tumor cell mobility by degradation of extracellular matrix. We therefore examined CNI-1493’s effect on M2-induced protease activity. Gelatinase assays were performed with supernatants and cell lysates from tumor cells alone, polarized M1 and M2 macrophages, and co-cultures of the tumor cells and both types of macrophages. Gelatinase activity was below detectable levels in supernatants as well as lysates of all cells, which remained unchanged with CNI-1493 treatment (S3 Fig). This may indicate that protease inhibition is not the means through which CNI-1493 decreases M2-induced tumor cell invasion. Together, these results are consistent with a direct effect of the drug on the cross-talk between the tumor cells and the M2 macrophages that promotes an invasive behavior.

### CNI-1493 treatment significantly decreases metastatic Ewing Sarcoma tumor burden in a mouse model

**Fig 3:**
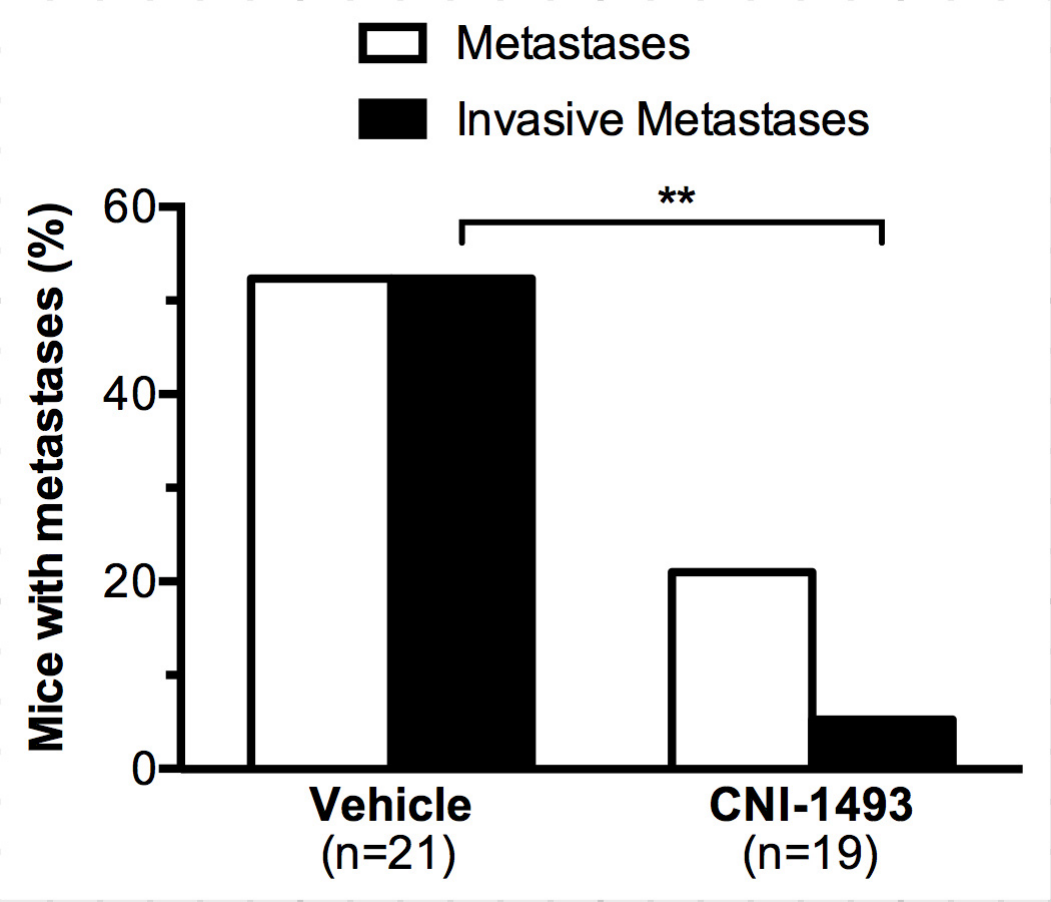
CNI-1493 reduces the incidence of invasive metastases. Nude mice were implanted with 10^6^ SK-NEP-1 cells in the left kidney and treated with intraperitoneal CNI-1493 (5 mg/kg/day) or vehicle for six weeks. In one replicate, drug and vehicle were delivered via subcutaneous osmotic pump placed at the time of surgery. In another replicate, drug was given via daily intraperitoneal injections. Mice were then sacrificed, lungs were sectioned, stained with hematoxylin and eosin, and examined for metastases with a pediatric pathologist in a blinded fashion. Immunohistochemistry with CD99 confirmed metastases. Metastatic foci were categorized based upon the presence of invasion into lung parenchyma. **p<0.01.

**Fig 4:**
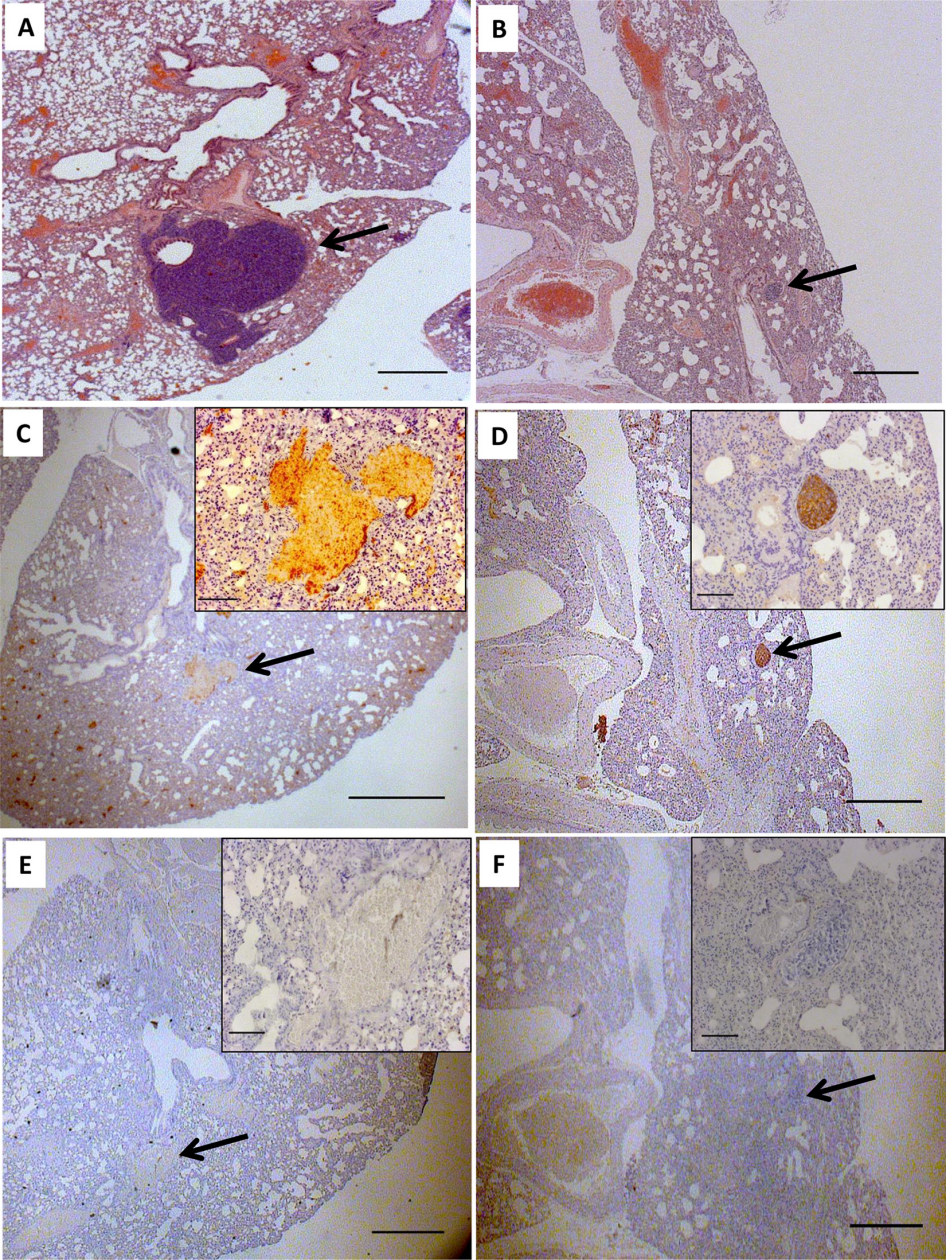
Lung sections of a metastatic Ewing Sarcoma mouse model demonstrate small, intravascular metastases in CNI-1493-treated mice. Mice were treated as in Fig 3. Sections from vehicle-treated mice (A, C, E) demonstrated large metastases invading the lung parenchyma. The mass in Fig 4A has been serially sectioned but was still visible in 4C. Sections from CNI-1493-treated mice (B, D,F) had smaller, non-invasive metastatic tumors that were confined to the pulmonary vasculature within the endothelium. Images are representative of typical metastases, arrows indicate metastatic tumor. A, B: Hematoxylin and eosin stain, bars represent 200 μm. C, D: Immunohistochemical staining for CD99, bars represent 200 μm. E,F: Negative control for CD99 staining, bars represent 200 μm. Inset bars represent 20 μm.

With the *in vitro* data showing that CNI-1493 inhibits tumor cell invasion through extracellular matrix, we utilized a well-established murine model of Ewing Sarcoma to examine CNI-1493’s potential anti-metastatic properties *in vivo* [68–71]. Nude mice were orthotopically implanted with SK-NEP-1 cells under the renal capsule. Similar to human disease, tumor cells in this model metastasize to the lung. CNI-1493 significantly inhibited invasive metastatic disease, and also suppressed total metastasis (Fig 3). Only four of 19 mice with primary tumor (21.1%) demonstrated any pulmonary metastases in the CNI-1493 treatment group, compared to 11 of 21 control animals (52.4%, p = 0.05). An invasive morphology was seen in only one treated animal, whereas all metastases in the control animals were invasive. This difference was highly significant (p<0.01). The histologic appearance of the metastatic tumors is shown in Fig 4. The control mice had large, highly invasive metastases (Fig 4A and 4C), while the metastases that did occur in the CNI-1493-treated mice were typically small and confined within the endothelium of the pulmonary vasculature (Fig 4B and 4D).

**Fig 5:**
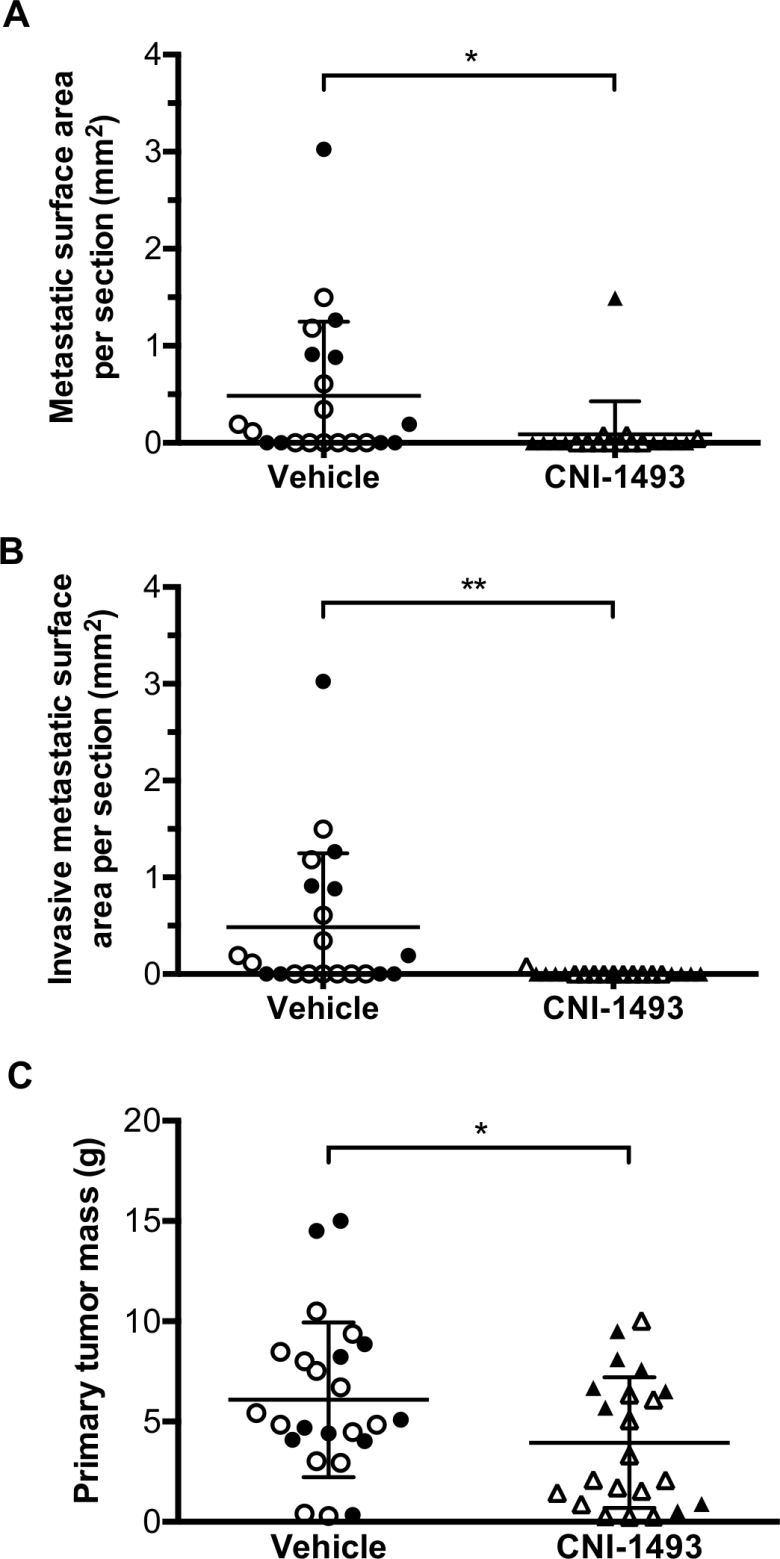
CNI-1493 significantly reduced metastatic tumor burden. Mice were treated as in Fig 3. Total cross sectional area of (A) any metastatic tumor, or (B) metastatic tumors demonstrating invasive morphology per tissue section was calculated for each mouse. (C) Primary tumors were excised at the time of animal sacrifice and weighed. Closed symbols represent the animal study replicate with intraperitoneal osmotic pump drug delivery, open symbols represent the animal study replicate with daily intraperitoneal injections. *p<0.05, **p<0.01, bars represent mean ± standard deviation.

The cross-sectional area of metastatic tumor per tissue section of each mouse with a primary tumor was used as a quantitative measure of metastatic tumor burden. Both total metastatic tumor burden (Fig 5A) and invasive metastatic tumor burden (Fig 5B) were significantly decreased in CNI-1493-treated mice. Control mice had a mean total metastatic cross sectional area of 0.49 ± 0.16 mm^2^ compared to 0.09 ± 0.08 mm^2^ for CNI-1493-treated mice (p<0.05). The mean invasive cross sectional area per mouse was 0.49 ± 0.16 mm^2^ in the control group and 0.004 ± 0.004 mm^2^ in the treated group (p<0.01).

There was no significant effect of CNI-1493 on establishment of primary tumors (86.4% for the treated group and 87.5% in the control group). Primary tumor mass was modestly but significantly decreased in CNI-1493-treated mice versus control mice, with a mean mass of 3.9 ± 3.3 g versus 6.1±3.9g, respectively (p<0.05, Fig 5C).

### CNI-1493 decreases M2-induced tumor cell invasion through an endothelium

**Fig 6:**
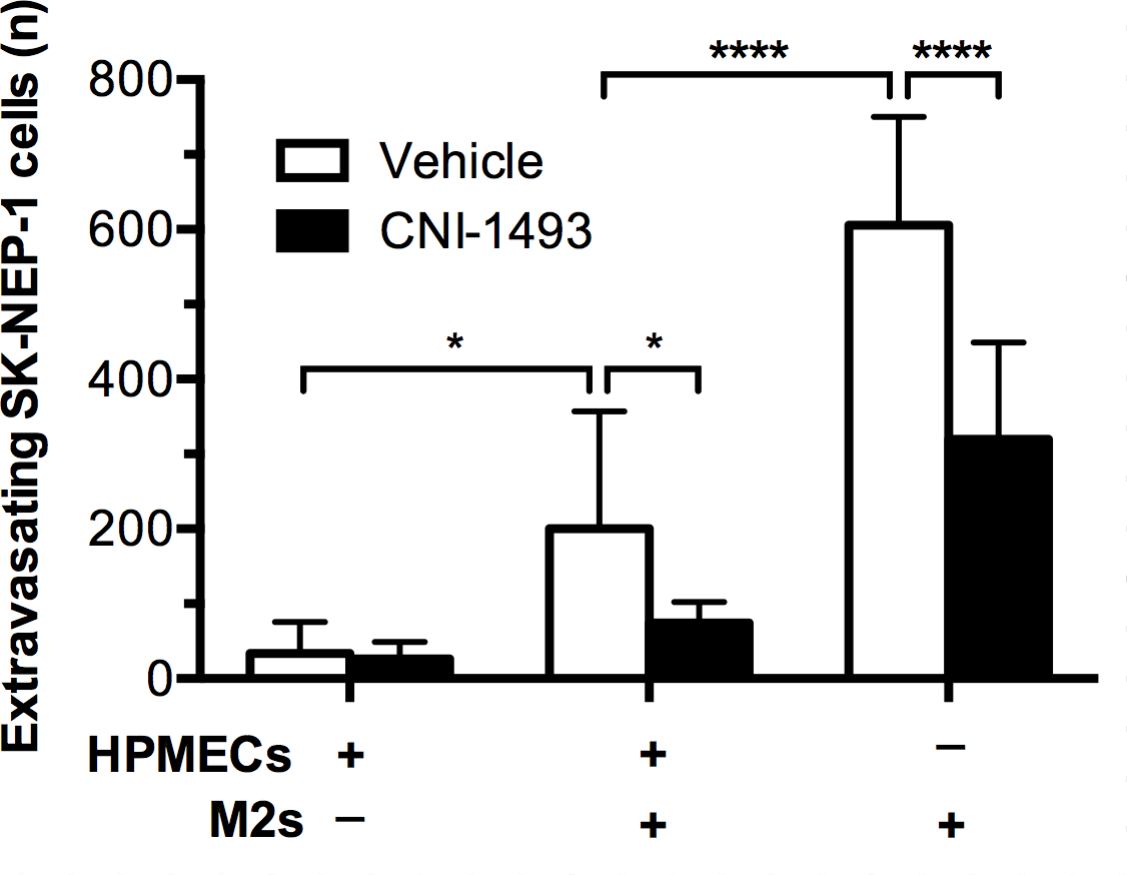
M2-induced tumor cell extravasation across an endothelial monolayer is inhibited by CNI-1493. GFP-expressing SK-NEP-1 cells were cultured alone or with M2 macrophages in the upper chamber of a transwell extravasation assay system, with or without the presence of an HPMEC monolayer. After 72 hours, the number of SK-NEP-1 cells invading into the lower chamber was counted. *p<0.05, ****p<0.0001. Bars represent mean ± standard deviation of four experiments.

Our observation that metastases in treated mice were intravascular suggested that CNI-1493 blocks extravasation. To further investigate this, we used an established *in vitro* extravasation assay [67], with the SK-NEP cells and M2 macrophages placed above a monolayer of human pulmonary microvascular endothelial cells (HPMECs) cultured on a thin layer of basement membrane extract coating the surface of the transwell inserts. This arrangement fundamentally replicates *in vitro* what metastasizing, extravasating tumor cells and associated macrophages would encounter within a pulmonary capillary *in vivo*: first the endothelium, followed by a basement membrane and ultimately the parenchyma. Light microscopy confirmed a monolayer of endothelial cells that was impermeable to Evan’s Blue dye (S2 Fig). The endothelial cells established a nearly complete barrier to invasion by the SK-NEP tumor cells, which was partially relieved by the presence of M2 macrophages (p<0.05, Fig 6). CNI-1493 suppressed the facilitated extravasation of tumor cells across the endothelial monolayer by 48.5% (95% CI 18.8–78.2%) compared to cultures without the drug. This proportional decrease by CNI-1493 treatment was not significantly different in the absence of an endothelial monolayer (45.0%, 95% CI 9.8–80.1%). This suggests that CNI-1493’s inhibition of extravasation is not mediated by a direct effect on the endothelial cells. The drug had no significant effect on the minimal extravasation of SK-NEP-1 cells cultured without macrophages.

## Discussion

In this study, we provided compelling evidence that treatment with the macrophage inhibitor CNI-1493 decreased metastasis in a mouse model of Ewing Sarcoma, in large part because of the inhibitory effect of the drug on extravasation. We demonstrated that tumor-supportive M2 macrophages greatly enhanced Ewing Sarcoma tumor cell invasion *in vitro* and that this was attenuated by treatment with CNI-1493.

Previous studies examining TAMs have largely focused on tumor cell intravasation and the role of macrophages at the primary site, and only recently has attention been turned to macrophages’ influence at metastatic foci [11, 24, 67]. Our *in vitro* data underline that the endothelium serves as a mechanical barrier to tumor cell extravasation. M2 macrophages promoted tumor cells to cross this barrier, further supporting the established notion that tumor-supporting macrophages play a seminal function in the establishment of metastatic foci. Treatment with CNI-1493 impeded this critical step of malignant progression that would otherwise lead to more advanced disease.

In our animal model, CNI-1493 treatment yielded a modest reduction in the size of primary tumors. It is likely that this small decrease only partially accounts for the robust reduction in invasive metastases seen in the lung parenchyma. Inhibitory effects of CNI-1493 on intravasation at the primary site cannot be ruled out, but if egress from the primary site were the principal mechanism of action of CNI-1493, drug treatment would have resulted in fewer metastases with similar extents of invasion. However, we observed intravascular metastases with noninvasive morphologies in CNI-1493-treated mice, strongly suggesting that the primary effect of CNI-1493 consists of inhibiting extravasation.

To our knowledge, we provide the first comparison of tumor cell invasion stimulated by M2 versus M1 macrophages, and showed that only M2 macrophages induce invasion. Our findings are substantiated by other recent studies of sarcomas examining the role of macrophages in tumor cell invasion [26, 72]. Our data underscore the concept of pharmacologically targeting M2 macrophages as a means to manage metastatic cancer. CNI-1493 is a promising candidate to add to the growing list of such TAM-targeting agents [10, 27–34].

Our findings show that CNI-1493 decreases tumor cell invasion by acting through macrophages and not directly on tumor cells, as these results were not seen in the absence of macrophages. This is supported by previous studies demonstrating the specificity of CNI-1493 on cells of monocytic lineage [36, 45, 73].

The mechanism of CNI-1493’s inhibition of M2-stimulated tumor cell invasion and extravasation is not clear. Gelatinase activity was not detectable in tumor cells or macrophages, but the effects of CNI-1493 on degradation of various extracellular matrix components, such as proteoglycans, polysaccharides, and various proteins and families of collagen, warrants further investigation. Other possible mechanisms through which CNI-1493 could decrease tumor cell invasion and extravasation include inhibiting M2-induced tumor cell migration, disrupting tumor cell-macrophage communication, modifying actin cytoskeleton dynamics, and altering interactions with other stromal components of the tumor microenvironment and the endothelium. These will be the focus of future investigations.

The anti-metastatic effect of CNI-1493 in sarcoma is in concordance with the few studies examining the therapeutic effect of CNI-1493 in other cancers. In glioblastoma, CNI-1493 treatment substantially inhibits tumor cell invasion into surrounding brain tissue [45]. With strikingly similar results to our own, treatment of a breast cancer model with CNI-1493 results in a modest decrease in primary tumor size and a more substantial reduction in metastatic disease [46]. Thus, CNI-1493 has anti-invasive properties in a variety of tumor types.

These data are a compelling foundation for the treatment of local and metastatic Ewing Sarcoma with CNI-1493. If metastases can be prevented or confined to within the vasculature, we anticipate restricting disease progression and conferring a survival benefit to the patient. Questions remain about the impact of the small, intravascular metastases seen with CNI-1493 treatment on overall prognosis. Moreover, treating in the perioperative setting with CNI-1493 may reduce the seeding of tumor cells as a direct consequence of surgical excision, or could limit growth of micrometastatic disease once suppression signals from the primary tumor are lost [74]. These questions, as well as the issue of survival benefit of CNI-1493, will be addressed in the future with the development of a resection model of Ewing Sarcoma. Since few other models of this disease exist, a resection model is a needed tool to better understand how CNI-1493 may affect the course of Ewing Sarcoma [75]. As Ewing Sarcoma often manifests as a tumor of the bone, it also remains to be seen if results from this model will apply to models with a primary bone tumor.

Ultimately, patients with advanced Ewing Sarcoma succumb to metastatic disease. An agent that can limit metastatic disease is therefore necessary to improve survival, whether or not a patient initially presents with metastases. Immunomodulatory drugs that target TAMs are currently being developed and tested in the clinic to treat advanced disease. Given its low toxicity profile, our studies suggest the possibility of administering CNI-1493 as an anti-metastatic adjuvant agent to patients with local or advanced disease to prevent further metastatic dissemination and improve survival.

## Supporting Information

S1 ARRIVE Checklist NC3Rs ARRIVE Guidelines Checklist (fillable) pdf. (PDF)

S1 Fig Flow analysis of magnetically sorted peripheral blood mononuclear cells demonstrates >90% CD14^+^ monocytes. Human peripheral blood mononuclear cells were isolated from whole blood on a Ficoll gradient and enriched for monocytes by magnetically sorting for CD14^+^ cells. Cells were analyzed by flow cytometry for CD3 and CD14. Left image represents pre-sort population, right image represents post-sort population.
(TIFF)

S2 Fig HPMEC monolayer is impermeable to Evans Blue. Permeability of the combined basement membrane and HPMEC monolayer was assessed by Evan’s Blue diffusion after one hour. Bars represent mean ± standard deviation of replicate samples in one experiment.
(TIFF)

S3 Fig Gelatinase assays of cell lysates and concentrated supernatants. Cell lysates and concentrated supernatants were incubated with DQ-gelatin and fluorescence intensity measured. Serial dilutions of *Clostridium histolyticum* collagenase were used as standards, with the lowest detectable concentration represented in this image. Data are expressed as equivalents of *C*. *histolyticum* units.
(TIFF)

S1 Table Differentially expressed genes in polarized macrophages. Macrophages were polarized to M1 or M2 phenotypes and treated with 200 nM CNI-1493 or vehicle in three independent experiments. Genes are listed in top-to-bottom order as they appear in the heatmap of Fig 1B. Data represent fold relative to the mean expression of all samples for each gene, and are expressed as means of three independent experiments.
(TIF)
