# Supplementary figures and images for: The Macrophage Inhibitor CNI-1493 Blocks Metastasis in a Mouse Model of Ewing Sarcoma through Inhibition of Extravasation
Source: PLoS One. 2015 Dec 28;10(12):e0145197. doi: 10.1371/journal.pone.0145197 (PMC4692435; doi:10.1371/journal.pone.0145197)

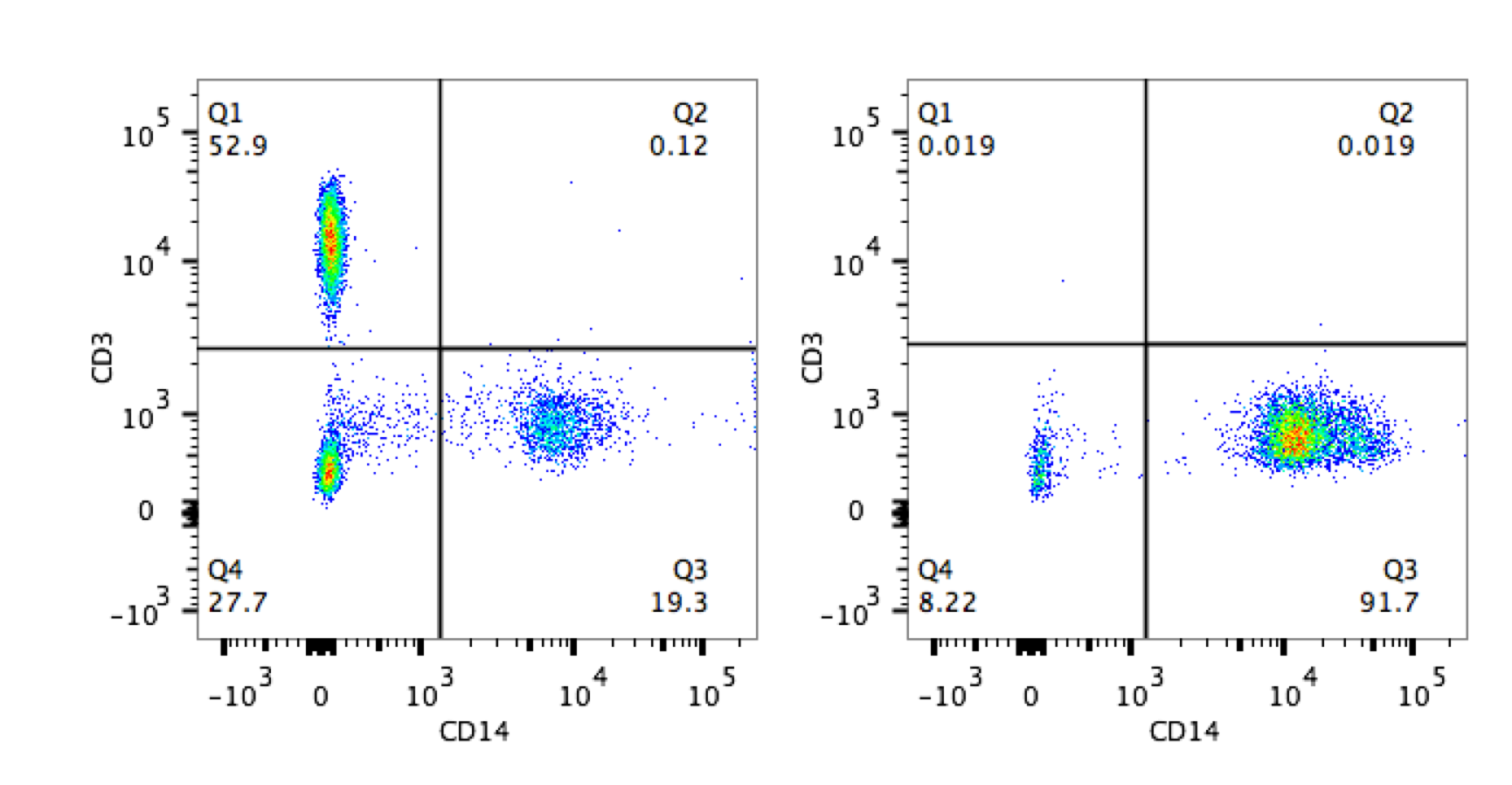

Supplement: S1 Fig — Human peripheral blood mononuclear cells were isolated from whole blood on a Ficoll gradient and enriched for monocytes by magnetically sorting for CD14+ cells. Cells were analyzed by flow cytometry for CD3 and CD14. Left image represents pre-sort population, right image represents post-sort population. (TIFF) [file pone.0145197.s002.tiff]

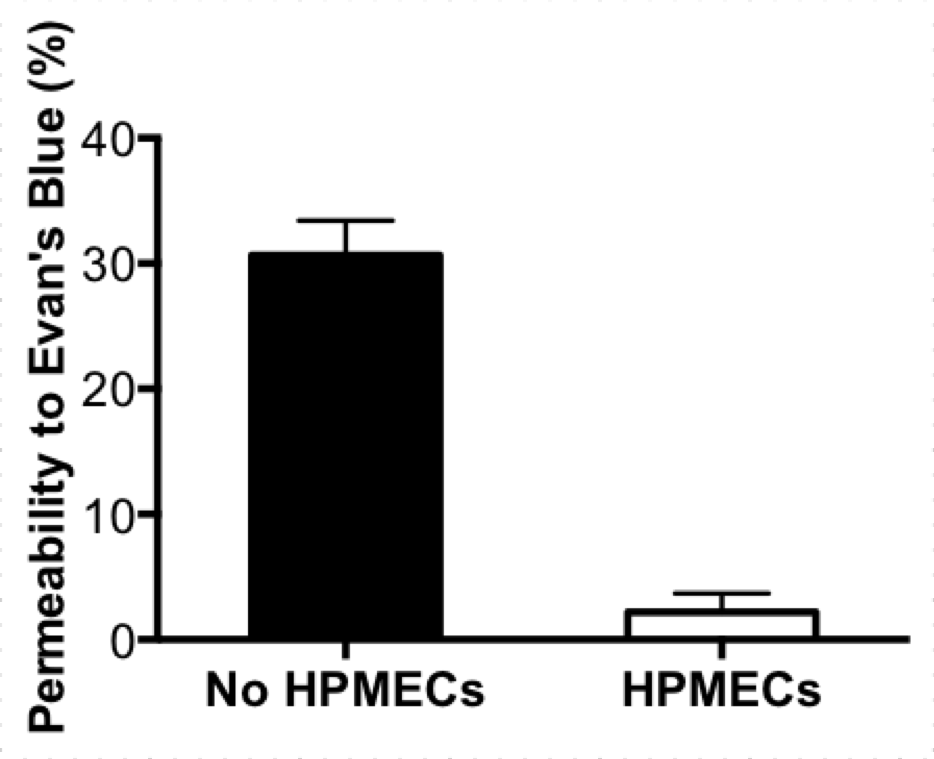

Supplement: S2 Fig — Permeability of the combined basement membrane and HPMEC monolayer was assessed by Evan’s Blue diffusion after one hour. Bars represent mean ± standard deviation of replicate samples in one experiment. (TIFF) [file pone.0145197.s003.tiff]

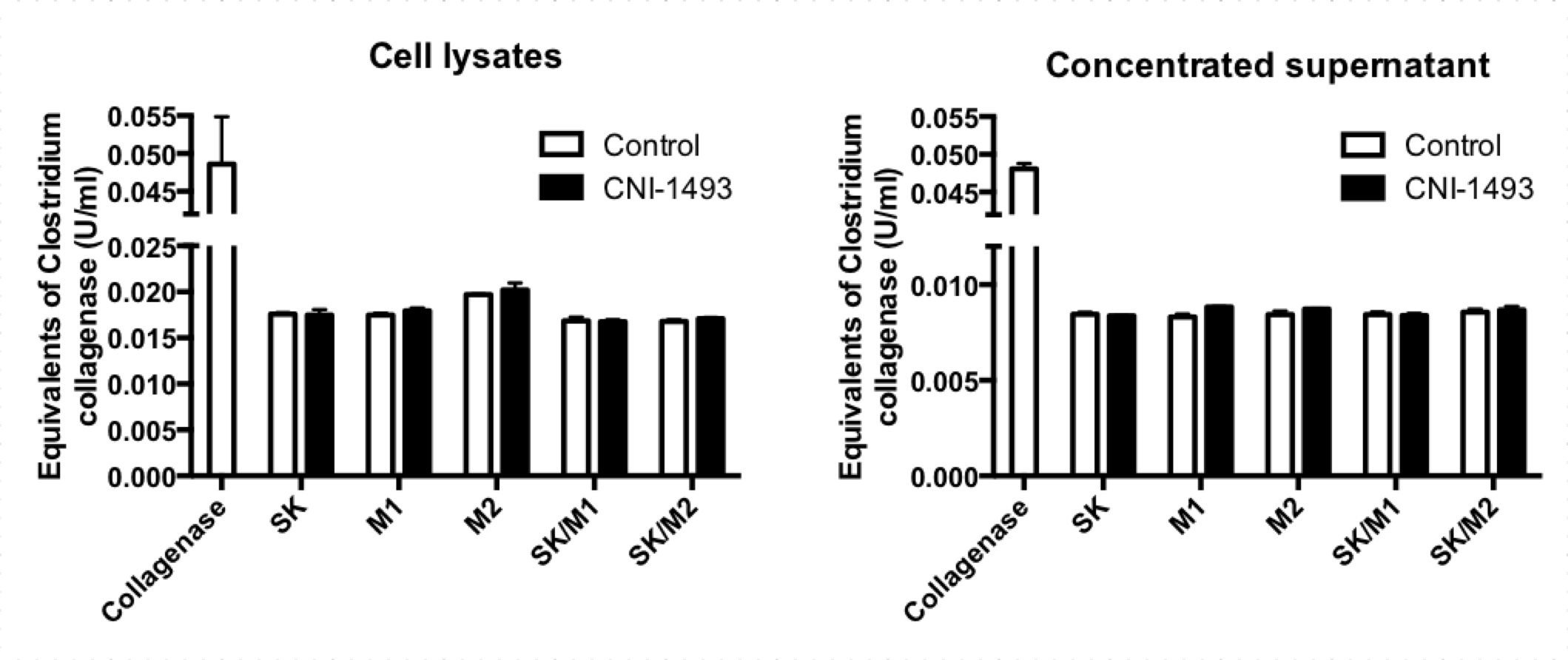

Supplement: S3 Fig — Cell lysates and concentrated supernatants were incubated with DQ-gelatin and fluorescence intensity measured. Serial dilutions of Clostridium histolyticum collagenase were used as standards, with the lowest detectable concentration represented in this image. Data are expressed as equivalents of C. histolyticum units. (TIFF) [file pone.0145197.s004.tiff]

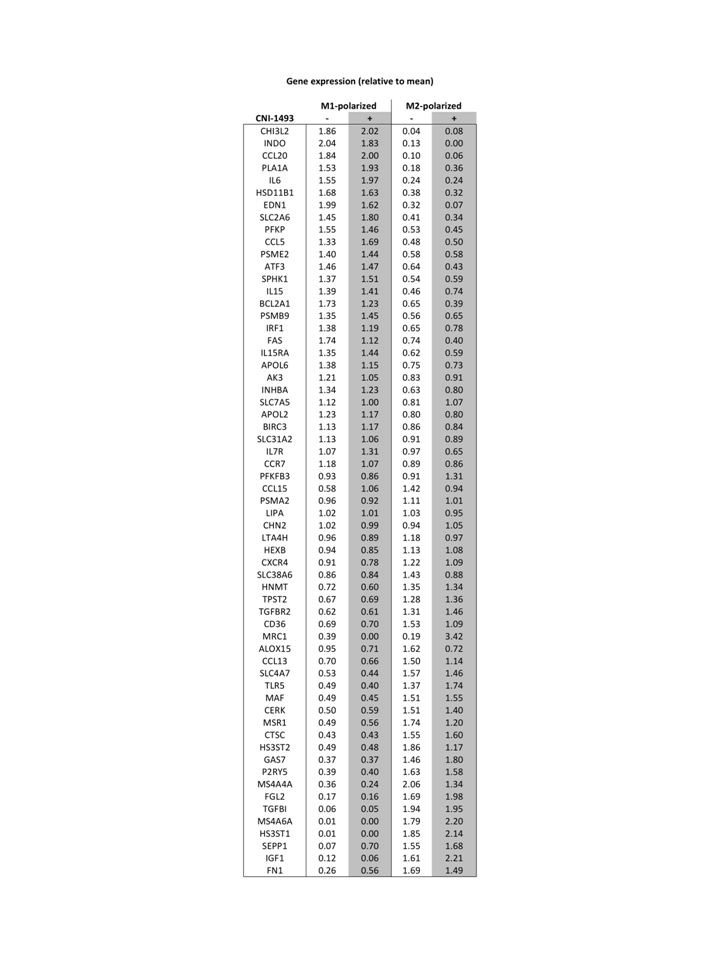

Supplement: S1 Table — Macrophages were polarized to M1 or M2 phenotypes and treated with 200 nM CNI-1493 or vehicle in three independent experiments. Genes are listed in top-to-bottom order as they appear in the heatmap of Fig 1B. Data represent fold relative to the mean expression of all samples for each gene, and are expressed as means of three independent experiments. (TIF) [file pone.0145197.s005.tif]
